# Evaluation of Clinical Characteristics of Critically Ill COVID-19 Patients With Renal Failure

**DOI:** 10.7759/cureus.62297

**Published:** 2024-06-13

**Authors:** Tunzala Yavuz, Omurhan Sarac, Hicret Yeniay, Yasemin Nadir, Ozcan Alpdogan

**Affiliations:** 1 Intensive Care Unit, Tepecik Training and Research Hospital, Health Sciences University, Izmir, TUR; 2 Anesthesiology and Critical Care, Izmir Bayraklı City Hospital, Izmir, TUR; 3 Infectious Disease, Tepecik Training and Research Hospital, Health Sciences University, Izmir, TUR

**Keywords:** acute kidney injury, covid-19, acute renal failure, creatinine, acute renal failure and hemodialysis in icu

## Abstract

Introduction: This study aimed to investigate the clinical characteristics and prognostic factors of critically ill COVID-19 patients with renal failure admitted to the ICU.

Methods: We analyzed 300 adult patients with SARS-CoV-2 infection admitted to the ICU between November 1, 2020, and June 1, 2022. Demographic data, renal function parameters, and outcomes were collected and analyzed.

Results: The median age was 72 years, and 54.3% were men. Mechanical ventilation was required for 86.3% of patients, with 71.0% needing invasive ventilation. Renal failure was present in 43.3% of patients at ICU admission, significantly associated with older age, higher mechanical and invasive ventilation needs, and increased ICU mortality (76.9% vs. 51.8%, p<0.001). Patients with renal failure had elevated levels of urea, creatinine, C-reactive protein (CRP), D-dimer, white blood cell (WBC), neutrophil (Neu), and procalcitonin (PCT) (p<0.001 for all).

Among patients with acute kidney injury (AKI), those with AKI had significantly higher median age (75 vs. 66 years, p<0.001), mechanical ventilation requirement (93.6% vs. 74.3%, p<0.001) and ICU mortality (79.1% vs. 35.4%, p<0.001). Elevated levels of urea (76 vs. 44 mg/dL, p<0.001) and creatinine (1.4 vs. 0.8 mg/dL, p<0.001), as well as inflammatory markers CRP and D-dimer (p=0.001), were observed in AKI patients.

Survivors had lower median age (66.0 vs. 74.0 years, p<0.001) and lower prevalence of chronic kidney disease (CKD) (4.5% vs. 12.8, p=0.019) and AKI (34.8% vs. 78.7%, p<0.001). Non-survivors exhibited higher levels of urea, creatinine, lactate dehydrogenase (LDH), CRP, ferritin, and D-dimer (p<0.001 for all).

Conclusion: Renal failure and AKI are prevalent in critically ill COVID-19 patients and are associated with worse outcomes. Elevated creatinine and urea levels at ICU admission are significant predictors of ICU mortality, underscoring the importance of early recognition and management of renal impairment in these patients.

## Introduction

COVID-19, caused by the SARS-CoV-2 virus, primarily targets the respiratory system but can also severely affect other organs, including the kidneys. The virus attaches to angiotensin-converting enzyme 2 (ACE2) receptors, which are predominantly found in the respiratory tract, leading to high viral loads and respiratory symptoms. ACE2 receptors are also present in other tissues such as renal epithelial cells, myocardial cells, enterocytes, and endothelial cells, contributing to extrapulmonary symptoms. The virus can exert a direct cytopathic effect on renal cells, triggering uncontrolled systemic inflammation that results in microangiopathy or hypercoagulability, leading to end-organ damage and subsequently the development of acute kidney injury (AKI) [1-5]. The systemic inflammation characteristic of severe COVID-19 can exacerbate pre-existing renal conditions and induce new-onset AKI through a combination of direct cytopathic effects and indirect mechanisms such as hypoxia and sepsis [1]. This multifactorial pathophysiology, as observed in significant pathological findings in the kidneys of deceased COVID-19 patients, indicates direct viral effects and widespread inflammation [1].

Patients may develop AKI on admission to intensive care or during hospitalization [6]. This condition significantly increases the risk of mortality through various mechanisms, including direct renal injury, cytokine storm, and systemic hypoxia [2,7]. The occurrence of severe AKI among critically ill COVID-19 patients admitted to the ICU is notably high. The presence of chronic kidney disease (CKD) further exacerbates the risk. The COVID-19 patients with pre-existing CKD had worse outcomes compared to those without CKD [7].

Studies have consistently shown a strong relationship between renal function at the time of admission to the intensive care unit (ICU) and mortality. Elevated creatinine levels, which indicate impaired renal function, have been significantly correlated with higher mortality in critically ill COVID-19 patients [3,8,9]. Understanding the relationship between renal function and COVID-19 severity is crucial for developing targeted interventions to reduce mortality. Early identification of AKI and timely initiation of renal replacement therapy (RRT) can significantly improve outcomes. Protocols are needed for the early identification of high-risk patients and the implementation of aggressive management strategies to prevent the progression of renal failure [2].

In our study, we aimed to investigate the clinical characteristics of critically ill COVID-19 patients with renal failure and to determine prognostic factors.

## Materials and methods

This retrospective study received approval from the local institutional Ethics Committee (approval no: 2022/12-03). It included all adult patients (age ≥ 18 years) admitted to our ICU between 1 November 2020 and 1 June 2022 with laboratory-confirmed SARS-CoV-2 infection, verified through a real-time reverse-transcriptase-polymerase-chain-reaction assay. Our patients received routine treatment based on the guidelines of the Ministry of Health [10].

Clinical and laboratory data

Demographic information, comorbidities, and laboratory data, such as complete blood count, renal function, coagulation parameters, and inflammatory markers (C-reactive protein (CRP), albumin, and ferritin), were recorded at ICU admission. Data collection also included the use of mechanical ventilation, RRT, discharge status, and mortality rates.

Patients admitted to the ICU may have pre-existing renal failure (chronic or acute) on admission or develop acute renal failure during their stay. Patients were screened for renal failure on admission to the ICU and throughout the ICU stay. The diagnosis of acute or chronic renal failure was determined according to the Kidney Disease Improving Global Outcomes (KDIGO) definition criteria. AKI was defined following the KDIGO criteria [11]. Hemodialysis (HD) or hemodiafiltration was applied to patients with RRT indication.

In order to examine the prognostic value of renal failure, the data of the patient group with renal failure were compared with the data of the patient group without renal failure.

Patients were divided into two groups, patients with AKI and without AKI during the hospital stay, and compared in terms of mortality and prognostic factors. In the next phase of the study, patients with AKI were divided into two groups (Group1: patients with AKI on ICU admission; Group2: patients who underwent AKI during hospitalization) and compared in terms of mortality and prognostic factors.

Outcome measures

The primary outcome was to identify the association between AKI and all-cause ICU mortality.

The secondary outcomes were the associations between AKI and ICU length of stay, hospital length of stay, and requiring mechanical ventilation.

Statistical analysis

The normality of the data distribution was evaluated using the Shapiro-Wilk test. Data are presented as mean ± SD for normally distributed variables and as median (interquartile range) or median (min-max) for non-normally distributed variables. Proportions were used as descriptive statistics for categorical variables. Independent group comparisons were performed using the two-tailed student’s t-test or the Mann-Whitney U test, depending on data distribution. The chi-square test was applied for categorical variable relationships, and the Kruskal-Wallis test was used for comparing means across multiple groups. Regression analyses were used to investigate the role of parameters impacting ICU mortality. A significance level of less than 0.05 was used to determine statistical significance. IBM SPSS version 27 (IBM Corp, Armonk, NY) was used as the statistical analysis program.

## Results

Study population

Three hundred adult patients with SARS-CoV-2 infection admitted to the ICU between 1 November 2020 and 01 June 2022 were included in this study. The median age among all patients was 72 years (19-98 years), and 163 (54.3%) were men. Among the patients, 259 (86.3%) required mechanical ventilation, 213 (71.0%) needing invasive mechanical ventilation. The main characteristics of the patients are presented in Table 1.

**Table 1:** Baseline and clinical characteristics of the patients HD: hemodialysis; MV: mechanical ventilation; ICU: intensive care unit; AKI: acute kidney injury

Variables	Patients (N=300)
Age, years, median(min-max)	72 (19-98)
Gender, men n(%)	163 (54.3%)
Coexisting Disorders	
Arterial hypertension, n(%)	155 (51.7%)
Diabetes mellitus, n(%)	102 (34%)
Chronic respiratory disease, n(%)	52 (17.3%)
Coronary artery disease, n(%)	73 (24.3%)
Heart failure	41 (13.7%)
Cerebrovascular disease, n(%)	34 (11.3%)
Chronic kidney disease, n(%)	29 (9.7%)
Routine HD, n(%)	14 (4.7%)
Received MV, n(%)	259 (86.3%)
Received invasive MV, n(%)	213 (71.0%)
Renal failure at ICU admission, n(%)	130 (43.3%)
Needing HD, n(%)	68 (22.7%)
HD applied, n(%)	33 (11%)
AKI at ICU admission, n(%)	114 (38%)
ICU length of stay, median(min-max)	9 (1-45)
Hospital length of stay, median(min-max)	15 (1-188)
ICU mortality, n(%)	188 (62.7%)

Renal failure

At the time of ICU admission, 130 (43.3%) of 300 patients had a diagnosis of renal failure (acute and/or chronic). Specifically, 29 patients (9.7%) had a diagnosis of CKD and 14 (4.7%) were undergoing routine HD. Patients with renal failure at admission were significantly older (median age 77 years), required more mechanical and invasive ventilation, and had higher ICU mortality rates compared to those without renal failure. Specifically, 93.1% of patients with renal failure required mechanical ventilation vs. 81.2% without renal failure (p=0.002), and 80.8% vs. 63.5% required invasive mechanical ventilation (p=0.001). ICU mortality was significantly higher in the renal failure group (76.9% vs. 51.8%, p<0.001). RRT was needed in 52.1% of patients with renal failure compared to 18.8% without (p<0.001), and 38.5 of those with renal failure receiving HD compared to 10.6% without (p<0.001). Patients with renal failure showed significantly elevated levels of urea, creatinine, CRP, D-dimer, white blood cell (WBC), neutrophil (Neu), and procalcitonin (PCT) (p<0.001 for all) (Table 2).

**Table 2:** Clinical and laboratory characteristics among patients with and without renal failure on admission ICU: intensive care unit; MV: mechanical ventilation; IMV: invasive mechanical ventilation; AKI: acute kidney injury; CKD: chronic kidney injury; RRT: renal replacement therapy; HD: hemodialysis; LDH: lactate dehydrogenase; CRP: C-reactive protein; WBC: white blood cell count; PCT: procalcitonin Note: All laboratory values taken at the time of admission, p values obtained from the chi-square test, or Mann-Whitney U test.

Variables	Without Renal Failure on Admission (N=170)	With Renal Failure on Admission (N=130)	U-value	Z-value	X^2^ value	df	P-value
Age, years, median (IR)	67.5 (27)	77 (19)	8057.0	˗4.021	-	-	<0.01
ICU length of stay, median(min-max)	9 (1-41)	9 (1-45)	10364.0	˗0.922	-	-	0.357
Hospital length of stay, median (min-max)	15 (1-188)	14(1-107)	9757.0	˗1.737	-	-	0.082
Received MV, n(%)	138 (81.2%)	121 (93.1%)	-	-	8.842	1	0.003
Received IMV, n(%)	108 (63.5%)	105 (80.8%)	-	-	10.634	1	0.001
ICU mortality, n(%)	88 (51.8%)	100 (76.9%)	-	-	19.930	1	<0.001
AKI, n	0	114	-	-	-	-	-
CKD, n	0	29	-	-	-	-	-
Needing RRT, n (%)	32 (18.8%)	69 (52.1)	-	-	38.703	1	<0.001
HD applied, n(%)	18 (10.6%)	50 (38.5)	-	-	41.006	2	<0.001
Albumin (g/dL)	2.85 (1.4-4)	2.8 (1.9-3.7)	9966.0	˗1.244	-	-	0.213
Urea (mg/dL)	46 (8-139)	115 (48-270)	1664.5	˗12.606	-	-	<0.001
Creatinine (mg/dL)	0.9 (0.3-1.6)	2.0 (1.2-6.3)	126.00	˗14.696	-	-	<0.001
LDH (IU/L)	395 (134-1993)	383 ( 129-1340)	10532.0	˗0.274	-	-	0.784
CRP (mg/dL)	102.8 (2.9-420)	118.5 (1.7-553)	8479.5	˗3.452	-	-	<0.001
Ferritin (µg/L)	416.5 (15-2675)	400 (27-9742)	6696.0	˗0.756	-	-	0.450
D-dimer (µg/L)	1445 (190-30310)	2810 (190-35200)	7883.5	˗3.838	-	-	<0.001
Fibrinogen (mg/dL)	466 (155-7656)	466 (80-900)	9601.5	˗1.143	-	-	0.253
WBC (x10∧3/uL)	9.5 (1.3-26.9)	10.4 (0.7-48)	9048.5	˗2.688	-	-	0.007
Neutrophil (x10∧3/uL)	8 (0.8-25.4)	9.2 (0.5-42.8)	8977.5	˗2.784	-	-	0.005
Lymphocyte (x10∧3/uL)	0.6 (0.1-3.4)	0.5 (0.1-2.9)	9922.5	˗1.519	-	-	0.129
Hemoglobin (g/dL)	11.1 (6.7-16.1)	10.3 (6.5-16.6)	8311.5	˗3.679	-	-	<0.001
Platelet count (x10∧3/uL)	214 (7-627)	175 (10-570)	9133.5	˗2.574	-	-	0.01
PCT (µg/L)	0.17 (0.01-45.2)	0.73 (0.06-290)	4732.5	˗8.418	-	-	<0.001

Acute kidney injury

The study compared patients with and without AKI. Notably, AKI patients were significantly older (median age 75.0) compared to those without AKI (median age 66.0, p<0.001). AKI patients had a higher rate of receiving mechanical ventilation (93.6% vs. 74.3%, p<0.001). The need for invasive mechanical ventilation was also significantly higher in AKI patients (83.4% vs. 50.4%, p<0.001). ICU mortality was higher in AKI patients (79.1% vs. 35.4%, p<0.001). Patients with AKI had significantly higher levels of urea (76 vs. 44 mg/dL, p < 0.001) and creatinine (1.4 vs. 0.8 mg/dL, p < 0.001). Inflammatory markers such as CRP (110.5 vs. 105 mg/L, p = 0.001) and D-dimer (2530 vs. 1580 ng/mL, p = 0.001) were also elevated in the AKI group. Ferritin levels did not differ significantly between the groups (p = 0.240) (Table 3).

**Table 3:** Clinical and laboratory characteristics among AKI and non-AKI ICU: intensive care unit; MV: mechanical ventilation; IMV: invasive mechanical ventilation; AKI: acute kidney injury; CKD: chronic kidney injury; RRT: renal replacement therapy; HD: hemodialysis; LDH: lactate dehydrogenase; CRP: C-reactive protein; WBC: white blood cell count; PCT: procalcitonin Note: AKI stage and all laboratory values taken at the time of admission, p values obtained from the chi-square test, or Mann-Whitney U test

Variables	Non-AKI (N=113)	AKI (N=187)	U-value	Z-value	X^2^ value	df	P-value
Age, median (IR)	66.0 (32)	75.0 (20)	7158.0	˗4.682	-	-	<0.001
ICU length of stay, median (min-max)	9 (2-37)	13 (2-41)	8671.5	˗2.605	-	-	0.009
Hospital length of stay, median (min-max)	15 (4-157)	17 (2-188)	10078.0	˗0.670	-	-	0.503
Received MV, n(%)	84 (74.3%)	175 (93.6%)	-	-	22.114	1	<0.001
Received IMV, n(%)	57 (50.4%)	156 (83.4%)	-	-	37.209	1	<0.001
ICU mortality, n(%)	40 (35.4%)	148 (79.1%)	5943.5	˗7.578	57.616	1	<0.001
AKI STAGE: 1	-	56	-	-	-	-	-
AKI STAGE: 2	-	24	-	-	-	-	-
AKI STAGE: 3	-	21	-	-	-	-	-
Needing HD, n	13	88	-	-	39.871	1	<0.001
HD applied, n	12	56	-	-	41.892	2	<0.001
Albumin (g/dL)	2.9 (1.5-4)	2.8 (1.4-3.7)	9346.5	˗1.536	-	-	0.125
Urea (mg/dL)	44.0 (8-187)	76 (15-270)	4585.0	˗8.215	-	-	<0.001
Creatinine (mg/dL)	0.8 (0.3-4.4)	1.4 (0.4-6.3)	4857.5	˗7.853	-	-	<0.001
LDH (IU/L)	382 (134-1601)	392.5 (129-1993)	9725.5	˗0.810	-	-	0.418
CRP (mg/L)	105 (2.9-420)	110.5 (1.7-551.3)	8212.0	˗3.233	-	-	0.001
Ferritin (µg/L)	409 (15-5519)	420 (23-9742)	6307.5	˗1.176	-	-	0.240
D-dimer (µg/L)	1580 (190-12810)	2530 (190-30330)	7877.5	˗3.289	-	-	0.001
Fibrinojen (mg/dL)	479 (204-7656)	466 (80-900)	9237.5	˗1.105	-	-	0.269
WBC (x10∧3/uL)	9.1 (2.7-29.7)	10.4 (0.7-48)	9304.5	˗1.732	-	-	0.083
Neutrophil (x10∧3/uL)	7.8 (2.1-26.3)	8.9 (0.5-42.8)	9226.5	˗1.839	-	-	0.066
Lymphocyte (x10∧3/uL)	0.6 (0.1-3.4)	0.5 (0.1-3.4)	8762.0	˗2.485	-	-	0.013
Hemoglobin (g/dL)	10.9 (6.7-15.3)	10.6 (6.5-16.6)	9314.5	˗1.719	-	-	0.086
Platelet count (x10∧3/uL)	226 (7-579)	191.5 (10-627)	8429.0	˗2.935	-	-	0.003
PCT (µg/L)	0.2 (0.01-103)	0.3 (0.01-290)	7402.5	˗4.286	-	-	<0.001

Of the 187 patients with AKI, 114 presented with AKI on the day of admission in the ICU, with the remainder developing AKI during their ICU course.

Upon comparing patients who had AKI on day of the admission to the ICU (n=114) with those who developed AKI during ICU stay (n=73), we observed no statistically significant distinctions in terms of the requirement for mechanical ventilation, the necessity for HD, and ICU mortality rates. On the day of admission, as per KDIGO criteria, 57 patients had AKI stage 1, 27 patients had AKI stage 2, and 30 patients had AKI stage 3. Dialytic support was required for 68 (22.7% of all patients) and was applied for 33 (11%) (Table 4).

**Table 4:** Clinical and laboratory characteristics of the patients according to development of AKI (on admission or during hospitalization) ICU: intensive care unit; MV: mechanical ventilation; IMV: invasive mechanical ventilation; AKI: acute kidney injury; CKD: chronic kidney injury; RRT: renal replacement therapy; HD: hemodialysis; LDH: lactate dehydrogenase; CRP: C-reactive protein; WBC: white blood cell count; PCT: procalcitonin Note: All laboratory values taken at the time of admission, p values obtained from the chi-square test, or Mann-Whitney U test. P^X^ comparison between Group1:AKI on admission vs. Group2: AKI developed during hospitalization

Variables	Without Renal Failure (N=97)	Group1: AKI on Admission (N=114)	Group2: AKI Developed During Hospitalization (N=73)	U-value	Z-value	X^2^value	df	P^x^ value:
Age, median (IR)	64.5 (31)	77.0 (17)	73 (22)	3668.5	˗1.365	-	-	0.172
ICU length of stay, median (min-max)	7 (1-37)	9.5 (1-45)	12 (1-41)	3426.0	˗2.038	-	-	0.042
Hospital length of stay, median (min-max)	14 (1-157)	14 (1-107)	18 (1-188)	3463.5	˗1.933	-	-	0.053
Received MV, n(%)	69 (71.1%)	106 (93%)	69 (93.8%)	-	-	0.175	1	0.675
Received IMV, n(%)	44 (45.4%)	92 (80.7%)	64 (87.7%)	-	-	1.563	1	0.211
ICU mortality, n(%)	27 (27.8%)	87 (76.3%)	61 (83.6%)	-	-	1.416	1	0.234
Needing HD, n	0	56	32	-	-	0.499	1	0.480
HD applied, n	0	38	18	-	-	1.652	2	0.438
Albumin (g/dL)	2.95 (1.5-4)	2.75 (1.9-3.7)	2.8 (1.4-3.6)	4004.0	˗0.237	-	-	0.813
Urea (mg/dL)	34.5 (8-105)	114 (48-270)	54.5 (15-139)	799.5	˗9.310	-	-	<0.001
Creatinine (mg/dL)	0.8 (0.3-1.3)	2 (1.2-6.3)	0.9 (0.4-1.6)	89.5	˗11.289	-	-	<0.001
LDH (IU/L)	386.5 (134-1601)	384 (129-1340)	425 (150-1993)	4010.0	˗0.117	-	-	0.907
CRP (mg/L)	97.2 (2.9-420)	118 (1.7-553)	105.1 (18-344)	3468.5	˗1.918	-	-	0.55
Ferritin (µg/L)	401 (15-2240)	382 (32-9742)	450 (23-2675)	2518.0	˗0.644	-	-	0.520
d-dimer (µg/L)	1490 (190-12810)	3390 (190-35200)	1440 (330-30330)	3054.0	˗2.822	-	-	0.005
Fibrinogen (mg/dL)	472 (204-7656)	466 (80-900)	460 (155-890)	3889.5	˗0.256	-	-	0.798
WBC (x10∧3/uL)	9.2 (2.7-20.6)	11.1 (7-48)	10 (1.3-26.9)	3436.0	˗2.008	-	-	0.045
Neutrophil (x10∧3/uL)	7.8 (2.1-18.9)	9.7 (5-42.8)	8.3 (0.8-25.4)	3446.5	˗1.979	-	-	0.048
Lymphocyte(x10∧3/uL)	0.6 (0.1-3.4)	0.5 (0.1-2.9)	0.5 (0.1-3.4)	4067.5	˗0.260	-	-	0.795
Hemoglobin (g/dL)	11.5(6.7-15.3)	10.5 (6.5-16.6)	10.9 (8-16.1)	3266.5	˗2.478	-	-	0.013
Platelet count (x10∧3/uL)	240 (7-579)	173.5 (10-570)	201.5 (44-627)	3633.5	˗1.461	-	-	0.144
PCT (µg/L)	0.16 (0.01-21.4)	0.77 (0.06-290)	0.18 (0.01-45.2)	1994.0	˗5.943	-	-	<0.001

Survivors and non-survivors

The median age of non-survivors was significantly higher than that of survivors (74.0 years vs. 66.0 years, p < 0.001). A higher percentage of non-survivors had CKD compared to survivors (12.8% vs. 4.5%, p = 0.019), and AKI was notably more prevalent among non-survivors, with 78.7% experiencing AKI vs. 34.8% of survivors (p < 0.001). In our study, the number of survivors was 20 and the number of deaths was 51 in patients with AKI stage 1 on admission to the ICU. In stage 2, the number of survivors was four and the number of deaths was 23, and in stage 3, the number of survivors was six and the number of deaths was 26, with significantly different survival rates among patients. ICU mortality was significantly associated with the requirement for HD, with 46.3% of non-survivors needing HD compared to 12.5% of survivors (p < 0.001). All non-survivors required mechanical ventilation, with 97.3% receiving invasive mechanical ventilation.

The laboratory markers also differed significantly between survivors and non-survivors. Non-survivors had higher levels of urea (76 vs. 44 mg/dL, p < 0.001), creatinine (1.4 vs. 0.8 mg/dL, p < 0.001), lactate dehydrogenase (LDH) (440 vs. 317 U/L, p < 0.001), CRP (118 vs. 92.2 mg/L, p = 0.001), ferritin (450 vs. 280 ng/mL, p < 0.001), and D-dimer (2390 vs. 1465 ng/mL, p < 0.001) (Table 5).

**Table 5:** Clinical and laboratory characteristics among survivors and non-survivors ICU: intensive care unit; MV: mechanical ventilation; IMV: invasive mechanical ventilation; AKI: acute kidney injury; CKD: chronic kidney injury; RRT: renal replacement therapy; HD: hemodialysis; LDH: lactate dehydrogenase; CRP: C-reactive protein; WBC: white blood cell count; PCT: procalcitonin Note: AKI stage and all laboratory values taken at the time of admission, p values obtained from the chi-square test, or Mann–Whitney U test.

Variables	Survivors	Non-survivors	U-value	Z-value	X^2^ value	df	P-value
Patients, n (%)	112 (37.3%)	188 (62.7%)	-	-	-	-	<0.001
Age, median (IR)	66.0 (25)	74.0 (20)	7227.5	˗4.543	-	-	<0.001
Chronic kidney disease, n(%)	5 (4.5%)	24 (12.8%)	-	-	5.539	1	0.019
Routine HD	4	10	-	-	2.435	1	0.119
AKI yes, n(%)	39 (34.8%)	148 (78.7%)	-	-	57.616	1	<0.001
AKI STAGE 1-2-3	30	100	-	-	21.761	3	<0.001
ICU length of stay, median (min-max)	8 (2-40)	13 (2-41)	9048.0	˗2.039	-	-	0.041
Hospital length of stay, median (min-max)	18.5 (4-188)	15 (2-57)	8179.5	˗3.234	-	-	0.001
Received MV, n(%)	71 (63.4%)	188 (100%)	-	-	79.716	1	<0.001
Received IMV, n(%)	30 (26.8%)	183 (97.3%)	-	-	169.688	1	<0.001
Needing HD, n	14	87	-	-	35.855	1	<0.001
HD applied, n	11	57	-	-	36.332	2	<0.001
Albumin (g/dL)	2.9 (2-4)	2.8 (1.4-4)	8434.5	˗2.757	-	-	0.006
Urea (mg/dL)	44.0 (8-187)	76 (15-270)	5748.0	˗6.578	-	-	<0.001
Creatinine (mg/dL)	0.8 (0.3-4.4)	1.4 (0.4-6.3)	6834.5	˗5.090	-	-	<0.001
LDH (IU/L)	317 (134-1601)	440 (129-1993)	6878.0	˗4.797	-	-	<0.001
CRP (mg/L)	92.2 (1.7-553)	118 (8.3-394.4)	8142.0	˗3.283	-	-	0.001
Ferritin (µg/L)	280 (15-8138)	450 (20-9742)	5034.0	˗3.757	-	-	<0.001
D-dimer (µg/L)	1465 (210-10290)	2390 (190-30330)	7574.0	˗3.717	-	-	<0.001
Fibrinojen (mg/dL)	479 (216-7656)	466 (80-894)	9941.0	˗0.099	-	-	0.921
WBC (x10∧3/uL)	9.35 (2.5-37.8)	10.5 (0.7-48)	9751.0	˗1.069	-	-	0.285
Neutrophil (x10∧3/uL)	7.8 (2.1-33.7)	8.7 (0.5-42.8)	9614.5	˗1.257	-	-	0.209
Lymphocyte (x10∧3/uL)	0.6 (0.1-3.4)	0.5 (0.1-3.4)	8403.0	˗2.933	-	-	0.003
Hemoglobin (g/dL)	11.3 (6.5-16.1)	10.5 (7.1-16.6)	8986.0	˗2.122	-	-	0.034
Platelet count (x10∧3/uL)	234.5 (7-579)	187 (10-627)	7960.5	˗3.533	-	-	<0.001
PCT (µg/L)	0.18 (0.01-145)	0.3 (0.01-290)	7120.0	˗4.633	-	-	<0.001

## Discussion

The incidence of AKI in our ICU study was notably high at 62%, with patients who developed AKI experiencing significantly higher ICU mortality rates compared to those who did not (79.1% vs. 35.4%, p<0.001). Organ damage in COVID-19 patients including AKI, acute liver injury, and acute gastrointestinal injury, can result from the inflammatory response to SARS-CoV-2 and is linked to worse outcomes. AKI is common in critically ill COVID-19 patients and correlates with poorer prognosis and increased need for invasive respiratory support [4,12].

Renal failure

Renal failure in COVID-19 patients admitted to the ICU is associated with worse outcomes, including higher mortality rates and increased need for invasive respiratory support. These findings underscore the importance of integrating renal function management into the overall treatment plan for critically ill COVID-19 patients [12-14]. In our study patients with renal failure had a higher percentage of receiving ventilation (93.1% vs. 81.2%, p=0.001). This suggests that patients with renal impairment experience more severe respiratory problems and need more invasive treatment. Importantly, ICU mortality was significantly higher in the renal failure group (76.9% vs. 51.8%, p<0.001). Elevated biomarkers, such as urea, creatinine, CRP, and D-dimer, were more common in patients with renal failure, indicating a severe inflammatory response and renal impairment [2,15].

Non-AKI and AKI

Elderly patients often have diminished renal reserve and are more likely to have comorbid conditions that increase the risk of AKI. This observation is consistent with previous research indicating that older age is an important risk factor for developing AKI in critically ill patients [4,12,14]. The incidence of AKI in ICU varies between 19% and 80% in studies [4,11,12]. In our study, the incidence of AKI in ICU was 62%. Patients with AKI (including those diagnosed upon admission and those developing AKI during hospitalization) exhibit elevated rates of mechanical ventilation requirement, HD necessity, and mortality in comparison to patients without AKI. Studies have shown high mortality rates in critically ill COVID-19 patients with AKI), which is consistent with our results [2,4,5,12,13,16]. Higher mortality rates were found in the group of patients with COVID-19 and AKI (79.1%) compared to the group of patients without AKI (35.4%).

Elevated levels of urea, creatinine, CRP, ferritin, procalcitonin, LDH, and D-dimer are common in AKI and COVID-19 patients. High levels of these biomarkers indicate more severe renal dysfunction and an increased inflammatory response. Elevated CRP and D-dimer levels are markers of systemic inflammation and coagulopathy, both of which are known to contribute to poor outcomes in critically ill patients. Monitoring these markers is crucial for management and treatment [11,14,17-19].

The median age of non-survivors was significantly higher than that of survivors (74.0 years vs. 66.0 years, p < 0.001). A higher percentage of non-survivors had CKD compared to survivors (12.8% vs. 4.5%, p = 0.019), and AKI was notably more prevalent among non-survivors, with 78.7% experiencing AKI vs. 34.8% of survivors (p < 0.001). Since patients with CKD are immunosuppressed, many infections cause patients to stay in the hospital longer and may increase mortality [20,21]. In our study, we found a significant relationship between the presence of CKD and mortality in patients with COVID-19 infection (p = 0.019). ICU mortality (p<0.001) occurred in 62.7% (188) of all patients. ICU mortality was significantly associated with the requirement for HD, with 46.3% of non-survivors compared to 12.5% of survivors (p < 0.001). All non-survivors required mechanical ventilation, with 97.3% receiving invasive mechanical ventilation. The laboratory markers also differed significantly between survivors and non-survivors. Non-survivors had higher levels of urea (76 vs. 44 mg/dL, p < 0.001), creatinine (1.4 vs. 0.8 mg/dL, p < 0.001), LDH (440 vs. 317 U/L, p < 0.001), CRP (118 vs. 92.2 mg/L, p = 0.001), ferritin (450 vs. 280 ng/mL, p < 0.001), and D-dimer (2390 vs. 1465 ng/mL, p < 0.001). These findings support the theory that severe systemic inflammation and thrombotic events contribute significantly to mortality [14,18].

Increased creatinine levels in COVID-19 patients are associated with poor prognosis and high mortality. Cheng et al. found that elevated serum creatinine levels in COVID-19 patients significantly increased the need for intensive care and mortality rates [8]. Hirsch et al. revealed that high creatinine levels increase the risk of mortality in patients with COVID-19-related AKI [9]. Studies have shown that increased AKI stages are associated with higher mortality rates in critically ill patients with COVID-19 [22,23]. Our data also show that mortality increases with increasing AKI stage. These findings emphasize that creatinine levels are an important biomarker in the management of COVID-19 patients. High creatinine levels may reflect not only kidney damage but also overall disease severity. Therefore, in the follow-up of patients with COVID-19 infection who develop AKI, creatinine levels should be carefully monitored. In our study, creatinine levels and urea levels demonstrated a strong statistical association with ICU mortality (p<0.001), highlighting the severity of renal impairment in critically ill patients. Elevated creatinine and urea levels are a hallmark of renal dysfunction and are often associated with higher severity of illness and poorer prognoses. These findings are consistent with previous studies that have documented the prognostic value of creatinine and urea levels in predicting ICU mortality [8,9,13].

This study has several limitations. First, this study was retrospective. This means that some relevant data may have been missing or inaccurately recorded, which would affect data quality. It is difficult to establish causal relationships between renal failure, AKI, and patient outcomes in a retrospective study. Second, the small sample size may limit statistical power in analyzing certain subgroups. Third, the study was conducted in a single center, the demographic characteristics and treatment protocols of patients at this center may differ from other institutions, which may reduce the applicability of the findings to different settings or populations.

## Conclusions

This study highlights the significant impact of renal failure and AKI on the outcomes of critically ill COVID-19 patients admitted to the ICU. AKI presence is associated with higher mortality, longer ICU stays, and increased need for ventilatory support and HD. Elevated biomarkers such as urea, creatinine, CRP, and D-dimer on the day of admission underscore the severity of AKI and the increased mortality risk. These findings highlight the importance of early identification of renal complications to improve patient outcomes.

## References

[1] Peiris S, Mesa H, Aysola A (2021). Pathological findings in organs and tissues of patients with COVID-19: A systematic review. PLoS One.

[2] Ghosn M, Attallah N, Badr M (2021). Severe acute kidney injury in critically ill patients with COVID-19 admitted to ICU: Incidence, risk factors, and outcomes. J Clin Med.

[3] Doher MP, Torres de Carvalho FR, Scherer PF (2021). Acute kidney injury and renal replacement therapy in critically ill COVID-19 patients: risk factors and outcomes: A single-center experience in Brazil. Blood Purif.

[4] Kumthekar GV, Nagarkar MS, Purandare V, Shukla S, Yeravdekar R (2024). Clinical and biochemical characteristics of COVID-19-associated acute kidney injury (COVAKI): A proof-of-concept case-control study. Cureus.

[5] Sitina M, Sramek V, Helan M, Suk P (2023). Prognostic significance of early acute kidney injury in COVID-19 patients requiring mechanical ventilation: a single-center retrospective analysis. Ren Fail.

[6] Taher A, Alalwan AA, Naser N, Alsegai O, Alaradi A (2020). Acute kidney injury in COVID-19 pneumonia: A single-center experience in Bahrain. Cureus.

[7] Dirim AB, Demir E, Yadigar S (2021). COVID-19 in chronic kidney disease: A retrospective, propensity score-matched cohort study. Int Urol Nephrol.

[8] Cheng Y, Luo R, Wang K (2020). Kidney disease is associated with in-hospital death of patients with COVID-19. Kidney Int.

[9] Hirsch JS, Ng JH, Ross DW (2020). Acute kidney injury in patients hospitalized with COVID-19. Kidney Int.

[10] (2024). COVID-19 (SARS-CoV-2 ENFEKSİYONU) REHBERİ. https://hsgm.saglik.gov.tr/depo/Yayinlarimiz/Rehberler/covid-19rehberipdf.pdf.

[11] Thakkar J, Chand S, Aboodi MS (2020). Characteristics, outcomes and 60-day hospital mortality of ICU patients with COVID-19 and acute kidney injury. Kidney360.

[12] Lavrentieva A, Kaimakamis E, Voutsas V, Bitzani M (2023). An observational study on factors associated with ICU mortality in Covid-19 patients and critical review of the literature. Sci Rep.

[13] Kowalski KJ, Bhat S, Fedje M (2023). COVID-19 and kidney disease (KD): A retrospective investigation in a rural Southwestern Missouri Region patient population. Cureus.

[14] Kant S, Menez SP, Hanouneh M (2020). The COVID-19 nephrology compendium: AKI, CKD, ESKD and transplantation. BMC Nephrol.

[15] Panimathi R, Gurusamy E, Mahalakshmi S, Ramadevi K, Kaarthikeyan G, Anil S (2022). Impact of COVID-19 on renal function: A multivariate analysis of biochemical and immunological markers in patients. Cureus.

[16] Xu J, Xie J, Du B, Tong Z, Qiu H, Bagshaw SM (2021). Clinical characteristics and outcomes of patients with severe COVID-19 induced acute kidney injury. J Intensive Care Med.

[17] Al Abri SY, Burad J, Al Wahaibi MM (2023). The incidence of acute kidney injury (AKI) in critically ill COVID-19 patients: A single-center retrospective cohort study at a tertiary level hospital in Oman. Cureus.

[18] Yavuz T, Orhan S, Rollas K (2023). Evaluation of clinical features and laboratory findings in critical intensive care unit patients with severe coronavirus disease-19 who underwent extracorporeal cytokine adsorption. Ther Apher Dial.

[19] Celik D, Inceer O (2023). Evaluation of the effectiveness of NLR, LMR, PLR, d-NLR, LeCR, LCR, NMR bioparameters in the course of COVID-19. Abant Med J.

[20] Jdiaa SS, Mansour R, El Alayli A, Gautam A, Thomas P, Mustafa RA (2022). COVID-19 and chronic kidney disease: An updated overview of reviews. J Nephrol.

[21] Khan BA, Tagore R, Rastogi S (2023). The impact of COVID-19 infection control measures on end-stage renal disease patients in a community hemodialysis setting. Cureus.

[22] Schaubroeck H, Vandenberghe W, Boer W (2022). Acute kidney injury in critical COVID-19: A multicenter cohort analysis in seven large hospitals in Belgium. Crit Care.

[23] Bouguezzi N, Ben Saida I, Toumi R (2023). Clinical features and outcomes of acute kidney injury in critically ill COVID-19 patients: A retrospective observational study. J Clin Med.

